# Disrespect and abuse during labour and birth amongst 12,239 women in the Netherlands: a national survey

**DOI:** 10.1186/s12978-022-01460-4

**Published:** 2022-07-08

**Authors:** Marit S. G. van der Pijl, Corine J. M. Verhoeven, Rachel Verweij, Tineke van der Linden, Elselijn Kingma, Martine H. Hollander, Ank de Jonge

**Affiliations:** 1 Department of Midwifery Science, AVAG/Amsterdam Public Health, Amsterdam University Medical Centre (UMC), Vrije Universiteit Amsterdam, Van der Boechorststraat 7, 1081 BT Amsterdam, The Netherlands; 2 Department of Obstetrics and Gynaecology, Maxima Medical Centre, Veldhoven, The Netherlands; 3 Division of Midwifery, School of Health Sciences, University of Nottingham, Nottingham, UK; 4 Stichting Geboortebeweging (Birth Movement NL), Ede, The Netherlands; 5 Hechte Band, Boxtel, The Netherlands; 6 Department of Clinical Psychology, Faculty of Behavioural and Movement Sciences, VU University and Amsterdam Public Health Research Institute, Amsterdam, The Netherlands; 7 Department of Research and Innovation, GGzE Mental Health Institute, Eindhoven, The Netherlands; 8 Department of Philosophy, King’s College London, London, UK; 9 Amalia Children’s Hospital, Department of Obstetrics, Radboud University Medical Center, Nijmegen, The Netherlands; 10 Department of Midwifery Science, AVAG, The Amsterdam Reproduction and Development Research Institute, Amsterdam University Medical Centre (UMC), Vrije Universiteit Amsterdam, Amsterdam, The Netherlands

**Keywords:** Labour and birth, Intrapartum care, Childbirth experience, Birth trauma, Traumatic childbirth, Disrespect and abuse, Mistreatment, Respectful maternity care

## Abstract

**Background:**

Women experience disrespect and abuse during labour and birth all over the world. While the gravity of many forms of disrespect and abuse is evident, some of its more subtle forms may not always be experienced as upsetting by women. This study examines (1) how often women experience disrespect and abuse during labour and birth in the Netherlands and (2) how frequently they consider such experiences upsetting. We also examine (3) which respondent characteristics (age, ethnicity, educational level and parity) are associated with those experiences of disrespect and abuse that are upsetting, and (4) the associations between upsetting experiences of disrespect and abuse, and women’s labour and birth experiences.

**Methods:**

Women who gave birth up to five years ago were recruited through social media platforms to participate in an online survey. The survey consisted of 37 questions about experiences of disrespect and abuse divided into seven categories, dichotomised in (1) not experienced, or experienced but not considered upsetting (2) experienced and considered upsetting. A multivariable logistic regression analysis was performed to examine associated characteristics with upsetting experiences of disrespect and abuse. A Chi-square test was used to investigate the association between upsetting experiences of disrespect and abuse and overall birth experience.

**Results:**

13,359 respondents started the questionnaire, of whom 12,239 met the inclusion and exclusion criteria. Disrespect and abuse in terms of ‘lack of choices’ (39.8%) was reported most, followed by ‘lack of communication’ (29.9%), ‘lack of support’ (21.3%) and ‘harsh or rough treatment/physical violence’ (21.1%). Large variation was found in how frequently certain types of disrespect and abuse were considered upsetting, with 36.3% of women experiencing at least one situation of disrespect and abuse as upsetting. Primiparity and a migrant background were risk factors for experiencing upsetting disrespect and abuse in all categories. Experiencing more categories of upsetting disrespect and abuse was found to be associated with a more negative birth experience.

**Conclusions:**

Disrespectful and abusive experiences during labour and birth are reported regularly in the Netherlands, and are often (but not always) experienced as upsetting. This emphasizes an urgent need to implement respectful maternity care, even in high income countries.

**Supplementary Information:**

The online version contains supplementary material available at 10.1186/s12978-022-01460-4.

## Plain language summary

Disrespect and abuse during labour and birth is a globally recognized phenomenon and has been linked to traumatic birth experiences and PTSD. In our study, we investigated how often women experience disrespect and abuse during labour and birth in the Netherlands and what proportion of these experiences was found to be upsetting. We also looked at risk factors for experiencing upsetting disrespect and abuse and to what extent upsetting disrespect and abuse influences the overall labour and birth experience.

We conducted an online survey, with 12,239 respondents included in the analysis. We found a large variation in how frequently certain types of disrespect and abuse were considered upsetting, with 36.3% of women experiencing at least one situation of disrespect and abuse as upsetting. More subtle forms of disrespect and abuse, such as lack of choice, communication or support, were most prevalent and often considered upsetting. Giving birth for the first time and having a migrant background were risk factors for experiencing upsetting disrespect and abuse. Upsetting disrespect and abuse was found to have a strong impact on the overall labour and birth experience; with every additional experienced category of upsetting disrespect and abuse, the number of (very) positive labour and birth experiences decreases and the number of very negative ones increases.

Although disrespect and abuse is a complex issue and its measurement subjective, this study shows that there is still a long way to go before achieving optimal respectful maternity care for all women, even in high income countries.

**Supplementary Information:**

The online version contains supplementary material available at 10.1186/s12978-022-01460-4.

## Background

In addition to physical maternal and fetal health outcomes, women’s birth experiences are important indicators of good quality care [1]. Nevertheless, studies show that many women encounter disrespectful and abusive care during labour and birth, both in low and high-income countries. This can contribute to negative or even traumatic birth experiences [2, 3].

Disrespect and abuse -also referred to as ‘obstetric violence [4]’ or ‘mistreatment [2]’—during labour and birth, have multiple definitions. Freedman et al. (2014) defined it as follows: ‘*interactions or facility conditions that local consensus deems to be humiliating or undignified, and those interactions or conditions that are experienced as or intended to be humiliating or undignified’* [5]. It is identified as a global issue, caused by many factors, taking place with varying degrees of severity and in different contexts [2, 3]. Bohren et al. (2015) developed a typology of mistreatment in maternity care consisting of seven domains: physical abuse, sexual abuse, verbal abuse, stigma and discrimination, failure to meet professional standards of care, poor rapport between women and care providers, and health system conditions and constraints. This typology enables a categorization of disrespect and abuse that takes account of both the direct interaction between women and care providers, factors related to health care systems as well as broader influences that play a role in the occurrence of disrespect and abuse [2, 3].

Several studies provide insight into the alarmingly high prevalence of serious forms of disrespect and abuse in low and middle-income countries around the world, with percentages ranging from 33.3% in Mexico [6] to 71.0% in India [7]. In higher income countries disrespect and abuse are also common, but different subtypes take precedence, for instance through unbalanced information provision, lack of informed consent, coercion into medical procedures and dismissing birth plans, both in subtle and unsubtle ways [8, 9]. Thompson et al. (2014) studied information provision and informed consent during labour and birth among 3542 Australian women: 26.0% reported not being informed about risks and benefits and not being consulted about their episiotomies during labour and birth; 13.0% of women were not informed and not consulted about vaginal examinations [10]. Vedam et al. (2019) found that one in six women in the United States experienced mistreatment during labour and birth, and that maternal characteristics played an role in the level of experienced mistreatment: self-identification as non-white, and maternal age below 30 were found to be associated with experiencing higher levels of disrespect and abuse [11].

Little research exists on the occurrence of disrespect and abuse during labour and birth in the Netherlands. Several studies showed high satisfaction levels among Dutch women with perinatal care and the patient centeredness of care providers during labour and birth [12, 13]. At the same time, Stramrood et al. (2011) found that 9.1% of Dutch women experienced their birth as traumatic [14]. A survey among Dutch women with a traumatic birth experience showed that they most often attribute their traumatic experience to lack of control, communication issues and lack of support [15].

In 2016, ‘de Geboortebeweging’ (translated: ‘Birth Movement’), a Dutch client organization advocating for the rights of women in Dutch maternity care, initiated a campaign in which women were invited to share their negative experiences with maternity care online. This campaign was part of a global movement known as #breakthesilence, or #rosesrevolution, initiated in Spain in 2011 [16]. A content analysis of the shared experiences revealed that experiences involving ineffective communication, loss of autonomy and lack of consent were most commonly described as negative or traumatic [17]. This study suggested disrespect and abuse do take place during labour and birth in the Netherlands, but there is, as yet, no insight into their prevalence. There are also a question whether more subtle forms of disrespect and abuse, such as unbalanced information provision or lack of informed consent, are truly experienced as disrespectful and/or abusive by the majority of women; not all women desire elaborate information or wish to provide repeated active consent during labour and birth [18].

To address these gaps in knowledge, the current study examines (1) how often women experience disrespect and abuse during labour and birth in the Netherlands and (2) how frequently they consider such experiences upsetting. We also examine (3) which respondent characteristics (age, ethnicity, educational level and parity) are associated with those experiences of disrespect and abuse that are upsetting, and (4) the associations between upsetting experiences of disrespect and abuse, and women’s labour and birth experiences.

## Methods

### Study design and setting

In this cross sectional study, an online survey was conducted among women who had given birth up to five years previously in the Netherlands. Data collection took place between October 26 and December 17, 2020.

The Dutch maternity care system is divided into midwife-led and obstetrician-led care. Women with a low risk pregnancy receive midwife-led care from community midwives and have a choice to give birth either at home, in a birth centre, or in a hospital with their community midwife. Women with risk factors or complications in pregnancy or during labour are referred to a hospital where they receive obstetrician-led care from a team of hospital-based midwives, obstetric registrars and obstetricians [19]. In 2019, 50.0% of women who gave birth for the first time in the Netherlands started labour in midwife-led care, and 17.0% gave birth assisted solely by their primary care midwife. For multiparous women these numbers were 46.0% and 34.0%, respectively [20].

### Ethical approval and informed consent

Ethical approval was sought from the medical ethics committee of Amsterdam UMC. They confirmed that the Dutch Medical Research Involving Human Subjects Act (WMO) did not apply to this study. Therefore an official approval by the committee was not required (14th April 2020, reference: 2020.084). Respondents received information about the study on the webpage of the survey, after which they could start the questionnaire. Respondents could leave their email address at the end of the survey if they wanted to, (1) have a chance to win a gift card, and (2) remain informed about the results of the study. To secure the respondents’ privacy, collected email addresses were stored separately from the filled out questionnaires.

### Patient and public involvement

This study was initiated after the #breakthesilence campaign of the client organization Birth Movement in the Netherlands. Throughout the research design and process, two client representatives of the Birth Movement were involved as equal co-authors (RV & TL). They co-defined the research aims, co-designed the questionnaire, consulted their network during the data collection phase and contributed to writing the manuscript.

### Study population

Women who gave birth in the Netherlands between 2015 and 2020, who were at least 16 years old and able to understand the Dutch or English language, were included in the study. If a woman had given birth more than once during this time period, she was asked to fill out the questionnaire for her most recent birth only. Respondents who did not fill out any of the questions related to disrespect and abuse were excluded from analysis. The survey was available in Dutch and English. Women with reading or writing difficulties could contact the research team by telephone for assistance in filling out the questionnaire.

### Sampling techniques

The domain name https://baringervaring.nl/ (translated: childbirth experience) was registered and served as a home page for the study. This home page provided all necessary information to start the questionnaire in Dutch and in English. When women started the survey on the home page, they were transferred to an online survey software program (Survalyzer Nederland B.V, Utrecht, The Netherlands), where they could fill out the questionnaire.

We aimed for a large sample size over a recruitment period of 2 months. Special efforts were made to reach hard-to-reach groups. Respondents were recruited via social media with the help of social media influencers and professional and client organizations. 58 influencers who gave birth in the last five years were approached, of whom 16 agreed to help with disseminating the invitation to the questionnaire on a voluntary basis through the social media platform Instagram. The influencers varied in terms of age, ethnicity, educational level, parity, mode and location of birth and birth experience. Seventeen organizations representing hard-to-reach groups in society were approached through email or by telephone, of which nine organizations agreed to voluntarily assist in disseminating the invitation to the questionnaire through newsletters, email, Facebook or during live events. In all recruitment methods it was emphasized that it did not matter whether women experienced their latest birth as positive or negative; every birth experience is worthwhile (see Additional file 1 for more information on sampling techniques). Based on the pilot, we estimated the time needed to fill out the questionnaire would be 15–30 min.

### Measurement tools

The questionnaire was composed and extended in multiple feedback rounds by a project team consisting of client representatives, health care providers and researchers. The questionnaire was then piloted in three rounds among several client representatives and adjusted based on the feedback given. The pilot was used to establish face and content validity. The questionnaire was checked by a language monitor unit to secure the use of lay language and translated from Dutch to English by an official agency to secure high quality translation (See Additional file 2 for more information about the questionnaire development).

The questionnaire first contained factual questions about the pregnancy, birth, and personal characteristics. Then respondents were asked about their overall experience of labour and birth with the answer options: very positive, positive, negative and very negative/traumatic. The next section of the questionnaire contained 37 questions covering situations of disrespect and abuse, representing seven categories based on existing literature [2, 17, 21] and adapted to the Dutch context. The seven categories were: emotional pressure (three questions), unkindness/verbal abuse (four questions), harsh or rough treatment/physical violence (six questions), lack of communication (five questions), lack of support (five questions), lack of choices (seven questions) and discrimination (seven questions). Each question asked whether a particular situation/form of disrespect and abuse occurred during their labour and birth. The respondents could answer either *‘yes’* or *‘no’*. If the answer was yes, the respondent was asked: ‘*did you find this upsetting?’* to which the respondents could answer either ‘*yes I found it upsetting* or*’ no I did not find it upsetting’* (See Additional file 3 for the full list of questions).

### Data analysis

The data were imported into SPSS version 26 (IBM Corporation Inc. Armonk, NY, USA). Descriptive statistics of personal, pregnancy and birth characteristics were summarized and, where applicable, compared to the Dutch perinatal registry or general Dutch Statistics. An overview of all personal, pregnancy and birth characteristics included in the study can be found in Additional file 4.

Three analyses were conducted on the data. Firstly, the answers to 37 questions about disrespect and abuse, representing the seven categories, were presented with the use of descriptive statistics as: (1) ‘not experienced,’ (2) ‘experienced + not considered upsetting,’ and (3) ‘experienced + considered upsetting’. If a respondent had given a positive answer to at least one of the questions of the category, the overall category was scored as that the disrespect or abuse occurred.

Secondly, the categories were dichotomized into [A] ‘not experienced’, and ‘experienced + not considered upsetting’ (1, 2); and [B] ‘experienced + considered upsetting’ (3). Multiple imputation was applied to handle missing data for age, ethnicity and educational level [22]. Multivariable analysis was performed to evaluate the respondent characteristics (age, ethnicity, educational level and parity) associated with disrespect and abuse per category (regardless of the other categories). Pooled adjusted odds ratios (AOR) with 95% CI per category were calculated (p < 0.001). Odds ratios above one indicated higher odds for experiencing upsetting disrespect and abuse, compared to the reference group.

Lastly, the seven categories of disrespect and abuse were classified into the number of categories any particular respondent experienced as upsetting (0–7). The number of categories of disrespect and abuse was then stratified according to the overall labour and birth experience of the respondent: (1) *very positive*, (2) *positive*, (3) *negative*, and (4) *very negative/ traumatic*. A Chi-Square test of association was used to examine the association between the frequency of upsetting experiences of disrespect and abuse and the respondents’ overall birth experience (p < 0.05).

## Results

**Fig. 1:**
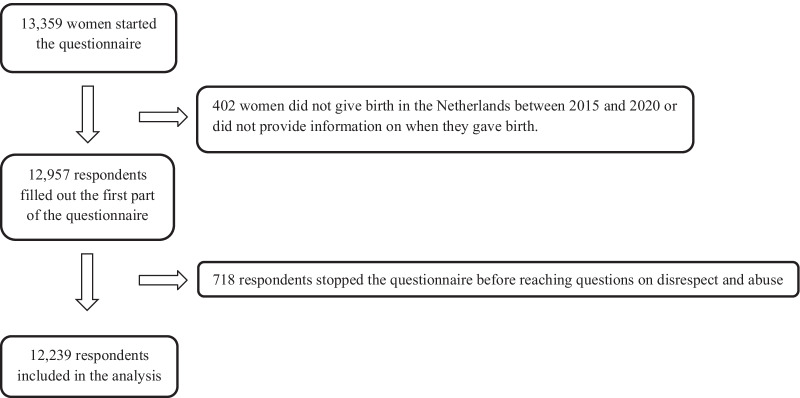
Flowchart of respondents included in analysis (n = 12,239)

In total, 13,359 respondents started the questionnaire, of whom 12,957 met the inclusion criteria. 718 respondents stopped the questionnaire before reaching the questions on disrespect and abuse, leaving 12,239 respondents available for analysis (Fig. 1). Filling out the questionnaire took the respondents 10–25 min, depending on the answers given. The respondents’ place of residence at time of birth based on postal codes, compared to the national data is visualized in Additional file 5.

Table 1 compares the characteristics of the respondents to Dutch national data. The largest group of respondents was between 30 and 34 years old at the time of giving birth (44.8%), and was of Dutch origin (respondent and both of her parents born in the Netherlands, 87.7%). Most respondents had a high educational level (69.6%). Of all respondents, 57.5% gave birth to their first child. All characteristics differ statistically significantly from the Dutch national data (p < 0.001).

**Table 1:** Respondent characteristics (age, ethnicity, educational level and parity) compared to National perinatal registry data, year 2019

Respondent characteristics		n (%) or mean [SD]		Chi-Square
		Respondents n = 12,239	National perinatal registry* or CBS**	p-value
Age at time of birth	Mean [standard deviation]	30,6 [3.97]		p < 0.001
	< 25	616 (5.6)	13,499 (8.4)*	
	25–29	3770 (34.3)	47,468 (29.4)	
	30–34	4924 (44.8)	64,390 (39.8)	
	35–39	1505 (13.7)	30,420 (18.8)	
	≥ 40	178 (1.6)	5844 (3.6)	
	Missing	1246		
Ethnicity	Both parents are born in the Netherlands	9514 (87.7)	3,158,000 (71.5)**	p < 0.001
	Respondent and (one of) her parents born abroad	384 (3.5)	762,000 (17.3)	
	Respondent born in NL, (one of) her parents born abroad	955 (8.8)	493,000 (11.2)	
	Missing	1386		
Educational level at time of birth	Low	673 (6.1)	975,000 (22.6)**	p < 0.001
	Middle	2659 (24.3)	1,710,000 (39.6)	
	High	7617 (69.6)	1,633,000 (37.8)	
	Missing	1290		
Parity	First birth	7033 (57.5)	71,950 (44.5)*	p < 0.001
	Second birth or more	5206 (42.5)	89,589 (55.5)	
	Missing	0		

*Based on women in the Netherlands who gave birth in 2019 registered by Perined (2020). Perinatale zorg in Nederland 2019 (n = 161,720)

**Based on women aged 15–55 in the Netherlands in 2019 registered by CBS Statistics Netherlands (n = 4,414,000)

Table 2 presents the pregnancy and birth characteristics of the respondents. Almost all women had a singleton pregnancy (98.0%). The majority of the respondents started their pregnancy in midwife-led care (84.6%). Almost two thirds had prepared a birth plan (64.2%). 60.0% of the respondents started labour in midwife-led care, of whom 44.0% were transferred to obstetrician-led care either during or immediately after birth. The largest group of respondents gave birth in the hospital in obstetrician-led care (64.3%). Almost three quarters (74.2%) of respondents had a spontaneous vaginal birth (with or without episiotomy) and 16.4% gave birth by caesarean section, of which the majority was unplanned (11.1%). Some form of pharmacological pain relief was used by 36.1% of the respondents. Almost one third of the births (31.7%) took place between March and December 2020, during the COVID-19 pandemic. Obstetric registrars and/or obstetricians were most often reported to be present at births (47.5%), followed by hospital based midwives (44.2%) and community midwives (42.8%). Almost all respondents (97.4%) reported having their partner present during birth.

**Table 2:** Pregnancy and birth characteristics of the respondents compared to National perinatal registry data, year 2019

Pregnancy and birth characteristics		n (%)		Chi-Square
		Respondents n = 12,239	National perinatal registry* n = 161,720	p-value
Singleton or multiple pregnancy	Singleton pregnancy	11,989 (98.0)	159,213 (98.5)	p < 0.001
	Multiple pregnancy	250 (2.0)	2477 (1.5)	
Responsible care provider at onset of pregnancy	Midwife-led care	10,347 (84.5)	138,498 (86.3)	p < 0.001
	Obstetrician-led care	1855 (15.2)	21,975 (13.7)	
	General practitioner	26 (0.1)		
	Other	11 (0.1)		
Planned place of birth one month prior to birth	Midwife-led care at home	3563 (29.1)		
	Midwife-led care in a birth centre	1209 (9.9)		
	Midwife-led care in a the hospital	4476 (36.6)		
	Obstetrician-led care in the hospital	2744 (22.4)		
	Hadn’t decided yet	247 (2.0)		
Birth plan prepared	Yes	7861 (64.2)		
	No	4378 (35.8)		
Responsible care provider at onset of labour	Midwife-led care	7342 (60.0)	77,801 (48.6)	p < 0.001
	Obstetrician-led care	4877 (39.8)	82,354 (51.4)	
	General practitioner	20 (0.2)		
	No care provider	2 (0.0)		
Onset of labour	Spontaneous	8204 (67.0)	104,906 (67.0)	p < 0.001
	Spontaneous rupture of membranes, followed by an induction with tablets or oxytocin	436 (3.6)	39,050 (25.0)	
	Rupture of membranes to induce labour	728 (5.9)		
	Induction of labour with tablets/foley catheter/oxytocin	2149 (17.6)		
	Caesarean section	722 (5.9)	12,460 (8.0)	
Unplanned transfer from midwife-led care to obstetrician-led care	Yes	3239 (44.0)	34,535 (44.4)	p < 0.398
	No	4123 (56.0)	43,266 (55.6)	
Mode of birth	Spontaneous vaginal birth	7613 (62.2)	118,823 (76.5)	p < 0.001
	Spontaneous vaginal birth with episiotomy	1463 (12.0)		
	Vacuum or forceps delivery	1156 (9.4)	11,240 (7.2)	
	Attempted vacuum or forceps, followed by a caesarean section	113 (0.9)	12,962 (8.3)	
	Unplanned caesarean section	1244 (10.2)		
	Planned caesarean section	650 (5.3)	12,460 (8.0)	
Pharmacological pain relief	No pharmacological pain relief	7451 (64.3)	93,334 (57.7)	p < 0.001
	Epidural	2619 (22.6)	68,386 (42.3)	
	Remifentanil	1100 (9.5)		
	Epidural and remifentanil	171 (1.5)		
	Other	200 (1.7)		
	Epidural and ‘other’	30 (0.3)		
	Remifentanil and ‘other’	6 (0.1)		
	Epidural, remifentanil and ‘other’	5 (0.0)		
	Missing	657		
Anesthesia during caesarean section	Spinal, epidural or combined spinal/epidural (CSE)	1839 (91.6)		
	General anesthesia	168 (8.4)		
Actual place of birth	Midwife-led care at home	2486 (20.3)	20,487 (12.7)	p < 0.001
	Midwife-led care at birth centre	480 (3.9)	4241 (2.6)	
	Midwife-led care at the hospital	1405 (11.5)	19,309 (11.9)	
	Obstetrician-led care at the hospital	7866 (64.3)	117,516 (72.7)	
	Other	2 (0.0)	155 (0.1)	
Gestational age at birth	< 37 + 0	723 (5.9)	10,030 (6.2)	p < 0.001
	37 + 0–38 + 6	2392 (19.6)	37,464 (23.2)	
	39 + 0–40 + 6	6410 (52.5)	85,341 (52.9)	
	41 + 0–41 + 6	2268 (18.6)	28,468 (17.7)	
	> 42 + 0	418 (3.4)	11 (0.0)	
	Missing	28		
Before or during COVID-19 pandemic	2015–February 2020 (prior COVID-19 pandemic)	8363 (68.3)		
	March–December 2020 (during COVID-19 pandemic)	3876 (31.7)		
Care providers present during birth	Community midwife	5236 (42.8)		
	Hospital based midwife	5408 (44.2)		
	Obstetric registrars and/or obstetrician	5759 (47.1)		
	Maternity care assistant	2997 (24.5)		
	Nurse	6684 (54.6)		
	Paediatrician	2121 (17.3)		
	Anaesthesiologist	1943 (15.9)		
	Care provider in training/student	2932 (24.0)		
	Other	68 (0.6)		
Other individuals present during birth	Partner	11,922 (97.4)		
	Other parent(s) of baby (non-partners)	16 (0.1)		
	Mother (in law)	902 (7.4)		
	Father (in law)	42 (0.3)		
	Child (ren)	81 (0.7)		
	Sister(s)	275 (2.3)		
	Brother(s)	11 (0.1)		
	Other family members	25 (0.2)		
	Friend(s)	164 (1.3)		
	Doula	127 (1.0)		
	Birth photographer	120 (1.0)		
	Others in care capacity	2 (0.0)		

*Based on women in the Netherlands who gave birth in 2019 registered by Perined (2020). Perinatale zorg in Nederland 2019

### Experienced (upsetting) disrespect and abuse during labour and birth

The answers to each question on disrespect and abuse are presented per category in Table 3. Overall, situations of disrespect and abuse were most often reported in the category ‘lack of choices’ (39.8%). Not being free to decide the position to give birth in was the situation most often indicated (25.3%), which a quarter of respondents found upsetting (27.8%). The least common (3%) but most frequently upsetting (93%) situation in this category was an intervention continued even the woman asked for it to be stopped. The second most common category was ‘lack of communication’, reported by 29.9% of the respondents, with variation in the extent to which these situations were experienced as upsetting (54.6–92.6%). ‘Lack of support’ was reported by 21.3% and was often experienced as upsetting (ranging from 78.2% to 92.9%). ‘Harsh or rough treatment/physical violence’ was experienced by 21.1% of the respondents, ranging from 52.9% upsetting (forced to stay in bed) to 98% upsetting (intervention experienced as sexual abuse). 4.6% of the respondents experienced a procedure as physical abuse, which 95.3% found upsetting, and 0.8% experienced a procedure as sexual abuse. ‘Unkindness/verbal abuse’ was reported by 10.1% of the respondents and these situations were often experienced as upsetting (ranging from 74.1% to 100%). Experienced situations of emotional pressure (3.0%) and discrimination (0.8%) were reported least. However, both categories had high levels of being considered upsetting: 84.3–95.4% for pressure and 82.4–100% for discrimination.

**Table 3:** Descriptive statistics of experienced disrespect and abuse during labour and birth presented per category and question

	Not experienced, n (% of total)	Experienced			Missing
		Total experienced, n (% of total)	Not upsetting, n (% of experienced)	Upsetting, n (% of experienced)	
**Emotional pressure**	**11,870 (97.0)**	**369 (3.0)**	**50 (13.6)**	**319 (86.4)**	
Were you threatened with bad test results or poor outcomes related to the health of your child?	11,915 (97.2)	324 (2.6)	51 (15.7)	273 (84.3)	0
Were threats made that involved withholding care from you or your child?	12,152 (99.3)	87 (0.7)	4 (4.6)	83 (95.4)	0
Were you threatened with legal consequences?	12,217 (99.8)	22 (0.2)	2 (9.1)	20 (90.9)	0
**Unkindness/verbal abuse**	**10,941 (89.9)**	**1226 (10.1)**	**193 (15.7)**	**1033 (84.3)**	
Did a health care provider say you were overreacting or you were pretending things were worse than they really were?	11,635 (95.6)	532 (4.4)	37 (7.0)	495 (93.0)	72
Were you subjected to insulting, harsh, unpleasant and/or derogatory comments?	11,499 (94.5)	668 (5.5)	64 (9.6)	604 (90.4)	72
Were you spoken to or shouted at in a harsh/rough or crude/coarse way?	11,658 (95.8)	509 (4.2)	132 (25.9)	377 (74.1)	72
Were you verbally abused?	12,149 (99.9)	18 (0.1)	0 (0.0)	18 (100.0)	72
**Harsh or rough treatment/physical violence**	**9648 (79.9)**	**2429 (21.1)**	**872 (35.9)**	**1557 (64.1)**	
Were you forced to stay in bed?*	9627 (84.7)	1745 (15.3)	822 (47.1)	923 (52.9)	867*
Were you forced into a particular position, or were you manually restrained?*	10,646 (93.6)	726 (6.4)	277 (38.2)	449 (61.8)	867*
Were you subject to rough physical treatment?	11,451 (94.8)	626 (5.2)	195 (31.2)	431 (68.8)	162
Were you slapped or kicked?	12,070 (99.9)	7 (0.1)	2 (28.6)	5 (71.4)	162
Was a (medical) intervention performed that you experienced as physical abuse?	11,526 (95.4)	551 (4.6)	26 (4.7)	525 (95.3)	162
Was a (medical) intervention performed that you experienced as sexual abuse?	11,977 (99.2)	100 (0.8)	2 (2.0)	98 (98.0)	162
**Lack of communication**	**8350 (70.1)**	**3562 (29.9)**	**1024 (28.7)**	**2538 (71.3)**	
Did you feel you were not being involved in the decision-making during labour and birth?	10,374 (87.1)	1538 (12.9)	699 (45.4)	839 (54.6)	327
Did you feel you were not being listened to?	10,773 (90.4)	1139 (9.6)	97 (8.5)	1042 (91.5)	327
Did you feel you were not being taken seriously?	10,649 (89.4)	1263 (10.6)	93 (7.4)	1170 (92.6)	327
Did you feel insufficiently at ease to ask questions?	11,278 (94.7)	634 (5.3)	86 (13.6)	548 (86.4)	327
Did you feel that you weren’t being given information that you should have been given?	9428 (79.1)	2484 (20.9)	664 (26.7)	1820 (73.3)	327
**Lack of support**	**9260 (78.7)**	**2498 (21.3)**	**362 (14.5)**	**2136 (85.5)**	
Did you feel you received too little attention, or were you left alone when you did not want to be left alone?	10,157 (86.4)	1601 (13.6)	208 (13.0)	1393 (87.0)	481
Did a health care provider refuse to assist you?	11,411 (97.0)	347 (3.0)	42 (12.1)	305 (87.9)	481
Did you ask for pain relief and was your request either ignored or refused by the care provider without there being a clear reason for this?	11,198 (95.2)	560 (4.8)	78 (13.9)	482 (86.1)	481
Were you or your partner denied (physical) contact with your child, without a clear reason?	11,518 (98.0)	240 (2.0)	17 (7.1)	223 (92.9)	481
Did you experience a lack of privacy? (for example during a physical examination)	11,156 (94.9)	602 (5.1)	131 (21.8)	471 (78.2)	481
**Lack of choices**	**6957 (60.2)**	**4602 (39.8)**	**2355 (51.2)**	**2247 (48.8)**	
Were you not free to decide who would be present at your delivery? (other than health care providers)**	7487 (94.9)	400 (5.1)	297 (74.3)	103 (25.7)	4352**
Did you feel compelled to accept care that you didn’t really want?	10,669 (92.3)	890 (7.7)	208 (23.4)	682 (76.6)	680
Were you not free to decide your position during contractions?*	8881 (81.1)	2076 (18.9)	1042 (50.2)	1034 (49.8)	1282*
Were you not free to decide the position in which you gave birth?***	5355 (74.7)	1816 (25.3)	1311 (72.2)	505 (27.8)	5068***
Were you told there were certain things you weren’t allowed to do, without there being a clear reason for this decision?	10,887 (94.2)	672 (5.8)	227 (33.8)	445 (66.2)	680
Was a (medical) intervention done without your having given clear permission in advance?	10,199 (88.2)	1360 (11.8)	708 (52.1)	652 (47.9)	680
Was a (medical) intervention continued even after you asked for it to be stopped?	11,214 (97.0)	345 (3.0)	24 (7.0)	321 (93.0)	680
**Discrimination**	**11,426 (99.2)**	**94 (0.8)**	**11 (11.7)**	**83 (88.3)**	
Did you experience discrimination based on race, ethnicity, cultural background, or language?	11,486 (99.7)	34 (0.3)	6 (17.6)	28 (82.4)	719
Did you experience discrimination based on age?	11,495 (99.8)	25 (0.2)	3 (12.0)	22 (88.0)	719
Did you experience discrimination based on sexuality and/or gender identity?	11,515 (100.0)	5 (0.00)	1 (20.0)	4 (80.0)	719
Did you experience discrimination based on physical or mental disability, illness or complaint?	11,510 (99.9)	10 (0.1)	0 (0.0)	10 (100.0)	719
Did you experience discrimination based on religion or belief?	11,496 (99.8)	24 (0.2)	2 (8.3)	22 (91.7)	719
Did you experience discrimination based on your appearance (other than racial appearance)?	11,514 (99.9)	6 (0.1)	1 (16.7)	5 (83.3)	719
Did you experience discrimination based on education, class, income or other socio-economic factors?	11,496 (99.8)	24 (0.2)	3 (12.5)	21 (87.5)	719

Bold headings report how many respondents experienced at least one situation in that category

*Respondents who had a planned caesarean section excluded

**Respondents who gave birth during COVID-19 pandemic excluded

***Respondents who had an epidural, vacuum extraction or a caesarean section excluded

### Associations between respondent characteristics and upsetting disrespect and abuse

Significant associations between the respondent’s age, ethnicity, educational level and parity, and upsetting experiences of disrespect and abuse are presented in pooled adjusted odds ratio’s per category in Table 4. Twenty imputed datasets were created to impute information [22]. Outcomes were compared to complete case analysis, which showed similar results. An overview of all outcomes including the non-significant pooled adjusted odds ratios and the odds ratios of the complete case analysis can be found in Additional file 6.

**Table 4:** The association between respondent characteristics and upsetting disrespect and abuse during labour and birth

Respondent characteristics		Pooled adjusted odds ratio [95% CI]*
**Emotional pressure**		
Ethnicity	Both parents are born in the Netherlands	1 (ref)
	Respondent and (one of) her parents born abroad	2.1 [1.3–3.3]**
	Respondent born in NL, (one of) her parents born abroad	**1.5 [1.0–2.1]**
Parity	First birth	1 (ref)
	Second birth or more	**0.65 [0.50–0.83]**
**Unkindness/verbal abuse**		
Ethnicity	Both parents are born in the Netherlands	1 (ref)
	Respondent and (one of) her parents born abroad	**1.5 [1.1–2.1]**
	Respondent born in NL, (one of) her parents born abroad	**1.4 [1.1–1.7]**
Parity	First birth	1 (ref)
	Second birth or more	**0.56 [0.48–0.64]**
**Harsh or rough treatment/physical violence**		
Higher age		**0.98 [0.97–0.99]**
Ethnicity	Both parents are born in the Netherlands	1 (ref)
	Respondent and (one of) her parents born abroad	**1.6 [1.3–2.1]**
	Respondent born in NL, (one of) her parents born abroad	1.2 [0.95–1.4]
Parity	First birth	1 (ref)
	Second birth or more	**0.56 [0.50–0.63]**
**Lack of communication**		
Higher age		**0.98 [0.97–0.99]**
Ethnicity	Both parents are born in the Netherlands	1 (ref)
	Respondent and (one of) her parents born abroad	**1.6 [1.2–2.0]**
	Respondent born in NL, (one of) her parents born abroad	1.2 [1.0–1.4]
Parity	First birth	1 (ref)
	Second birth or more	**0.47 [0.42–0.52]**
**Lack of support**		
Ethnicity	Both parents are born in the Netherlands	1 (ref)
	Respondent and (one of) her parents born abroad	**1.3 [1.0–1.7]**
	Respondent born in NL, (one of) her parents born abroad	**1.2 [1.0–1.4]**
Parity	First birth	1 (ref)
	Second birth or more	**0.48 [0.43–0.54]**
**Lack of choices**		
Higher age		**0.98 [0.97–0.99]**
Ethnicity	Both parents are born in the Netherlands	1 (ref)
	Respondent and (one of) her parents born abroad	**1.4 [1.1–1.8]**
	Respondent born in NL, (one of) her parents born abroad	1.2 [1.0-1.4]
Educational level	Low	1 (ref)
	Middle	1.1 [0.9–1.4]
	High	**1.3 [1.1–1.7]**
Parity	First birth	1 (ref)
	Second birth or more	**0.53 [0.48–0.59]**
**Discrimination**		
Ethnicity	Both parents are born in the Netherlands	1 (ref)
	Respondent and (one of) her parents born abroad	**5.9 [3.0–11.6]**
	Respondent born in NL, (one of) her parents born abroad	**3.4 [2–5.9]**
Parity	First birth	1 (ref)
	Second birth or more	**0.56 [0.34–0.93]**

*Adjusted odds ratio pooled over 20 imputed datasets. Pooled data was compared with complete case analysis, see Additional file 6

**Variables with at least 1 statistically significant result are presented in table. Significant adjusted odds ratios are shown in bold, with significance based on 2 decimals

Respondents with a migrant background had increased odds of experiencing upsetting disrespect and abuse compared to respondents who themselves, and both their parents, were born in the Netherlands. Odds ratios for all categories except for discrimination ranged from 1.2 to 2.1 (with a few of these not reaching significance). The odds ratios for the category discrimination were higher (AOR 3.4 and 5.9).

For all categories, respondents who reported their second birth or more had about half the odds of upsetting disrespect and abuse compared to respondents who gave birth for the first time (AOR varying between 0.47 and 0.56, with emotional pressure at 0.65).

With increasing age, the odds of experiencing upsetting disrespect and abuse decreased slightly for the categories physical violence, lack of communication and lack of choices (AOR 0.98).

Educational level only showed significant differences for lack of choices; highly educated respondents have increased odds of experiencing upsetting disrespect and abuse in this category compared to less educated respondents (AOR 1.3).

### Associations between upsetting disrespect and abuse and women’s overall labour and birth experiences

Table 5 reports the number of categories of disrespect and abuse respondents experienced, stratified for overall birth experience. In total, 79.1% of respondents reported a positive or very positive experience, 11.9% a negative and 9.0% a very negative or traumatic birth experience.

**Table 5:** Number of categories of disrespect and abuse respondents experienced as upsetting, in total and stratified for overall birth experience

	Total, n (%)	Very positive experience, n (%)	Positive experience, n (%)	Negative experience, n (%)	Very negative/Traumatic experience, n (%)
**Total population**	12,239*	**3924 (32.1)**	**5751 (47.0)**	**1459 (11.9)**	**1105 (9.0)**
No category	7338 (63.7)	3326 (45.3)	3543 (48.3)	311 (4.2)	158 (2.2)
One category	1605 (13.9)	307 (19.1)	887 (55.3)	262 (16.3)	149 (9.3)
Two categories	1071 (9.3)	97 (9.1)	555 (51.8)	257 (24)	162 (15.1)
Three categories	708 (6.1)	29 (4.1)	266 (37.6)	233 (32.9)	180 (25.4)
Four categories	423 (3.7)	9 (2.1)	114 (27.0)	152 (35.9)	148 (35.0)
Five categories	285 (2.5)	3 (1.1)	51 (17.9)	91 (31.9)	140 (49.1)
Six categories	70 (0.6)	2 (2.9)	7 (10.0)	19 (27.1)	42 (60.0)
All seven categories	20 (0.2)	0 (0.0)	2 (10.0)	3 (15.0)	15 (75.0)

Data in bold shows the overall birth experience of the total study population (n = 12,239)

*Number of respondents who filled out the question on overall experience differs from the number of respondents who filled out all 37 questions of the seven categories (n = 11,520)

In total, 54.4% of respondents reported at least one form of disrespect and abuse; 36.3% reported at least one form of upsetting disrespect and abuse. Of those experiencing no upsetting disrespect and abuse, over 90% had a positive/very positive experience. Of those experiencing one category of upsetting disrespect and abuse, 74.4% had a positive/very positive experience. Thereafter, the number of positive experiences steadily drops by about 10 to 15% for every additional category of upsetting disrespect and abuse. From three categories or more the percentage of negative/very negative birth experiences exceeded the number of positive/very positive ones.

Chi-square statistics showed a significant association between the number of categories of disrespect and abuse respondents experienced as upsetting, and their overall birth experience [χ^2^ (9, *n* = 11,520) = 3481.9, p < 0.001].

## Discussion

In this study, we investigated how often women experience disrespect and abuse during labour and birth in the Netherlands and what proportion of these experiences was found to be upsetting. Furthermore, we examined certain respondent characteristics which are associated with upsetting experiences of disrespect and abuse, as well as associations between upsetting disrespect and abuse and women’s overall labour and birth experience.

54.4% of respondents reported at least one form of disrespect and abuse. The categories ‘Lack of choices’ (39.8%) and ‘lack of communication’ (29.9%) were reported most. Considerable variation was found in how frequent disrespect and abuse were considered upsetting, ranging from 25.7% to 100%. In total, 36.3% experienced at least one situation of upsetting disrespect and abuse. Primiparity and a migrant background were risk factors for experiencing upsetting disrespect and abuse in all categories. There is a strong impact of experiencing disrespect and abuse on birth experience; with every additional experienced category of upsetting disrespect and abuse, the number of positive/very positive labour and birth experiences decreases and the number of very negative ones increases. This confirms previous findings about the impact of care providers’ actions and interactions [15, 23–25]. The 9.0% very negative or traumatic labour and birth experiences found in this study closely resembles the 9.1% of women reported to have had a traumatic birth experience in the Netherlands in 2011 [14]. This suggests that, despite a focus on respectful maternity care (RMC) provision in the last decade, [14, 15, 17] this number has not significantly improved.

A lack of choice was the category of disrespect and abuse most often reported by women. Not all women considered the situations in this category upsetting. This suggests not all women wish to be involved or mind care providers making decisions for them. However, it is also possible that women are not fully aware of their options, which limits their freedom of choice. At the same time a substantial portion of women did find situations of lack of choice upsetting, which is in line with previous studies showing a lack of choice can negatively affect women’s feeling of autonomy and control [17, 26, 27]. We should acknowledge that there are various ways and preferences in maintaining one’s autonomy; handing over decision capacity to a care provider could be one of them [28, 29]. This substantiates the need for an individualized approach in care provision during labour and birth, in which women experience enough room to express their personal preferences. Preferably, this conversation is already initiated by care providers in the antenatal period, so that women have time to think about their (expected) preferences prior to labour and birth.

Lack of communication was the second category most often reported, with almost all women experiencing these situations as upsetting. Effective communication is important, as it is essential for a positive birth experience and it enables access to information on the (health) status of both mother and baby during birth, which is directly relevant for exercising personal autonomy [30]. A lack of support by the caregiver was also often reported and experienced as upsetting, which underscores the necessity of continuous support for preventing a traumatic experience, also shown in previous research [15, 31, 32].

The above mentioned results are consistent with existing evidence showing that disrespect and abuse in high income countries are prevalent in more ‘subtle’ ways, compared to the abusive and violent behaviours more often reported in lower income countries [9]. However, this study shows that these ‘subtle’ ways are no less important: more than 90% of those feeling not being listened to or not being taken seriously, as well as being told they were overreacting, found this upsetting, compared to 70% for rough physical treatment. More severe forms of disrespect and abuse, such as being subjected to rough physical treatment or being forced in a certain position, might more often be related to emergency situations in which women have a higher tolerance for disrespectful care: the obviousness of the need for a procedure can help in understanding and thus experiencing the event as less upsetting [33].

Although situations of lack of choice, communication and support were most common, the other categories, including physical abuse, sexual abuse, verbal abuse, emotional pressure and discrimination were also all reported. That may seem striking in a modern health care setting such as the Netherlands, but is consistent with reports from other high income countries such as Spain, Italy and the United States [11, 34, 35].

The prevalence of occurrences of disrespect and abuse should not be mistaken for the prevalence of their intent; probably in the overwhelming majority of cases care providers do not intent the negative effects that women report. Care providers generally work hard for a healthy mother and baby, do not intent to cause harm, and may not be aware that their efforts can be interpreted as harmful [36]. Sometimes care providers judge a situation as urgent and may intervene without, in the eyes of the woman, sufficient communication or explanations, or care providers may assume knowledge or understanding that women do not possess [24]. That makes our results all the more important; precisely because care providers in almost all cases won’t intent the results that are reported here, it is important to realize they are, regularly, experienced as upsetting by woman.

The situations we report may also be facilitated by the health care system in which they occur. Often, there is a disproportionate focus on biomedical care in practice and education, with less attention being paid to women’s values and experiences [37]. The focus on medical outcomes is also present in society. There is a tendency to focus on the health of the baby, rather than the rights of the mother and her bodily integrity [30]. Furthermore, in the media, labour and birth are often medicalised and dramatised, mainly portraying the care provider as the central actor delivering during labour, instead of the woman [38]. This reflects the deeper dynamics of power and gender inequality in society that allow disrespect and abuse during labour and birth to occur [3]. Thus, disrespect and abuse are complex phenomena, with many aspects on an individual, health system and societal level [3]. A multi-faceted approach is required to tackle this issue.

Women with a migrant background reported upsetting disrespect and abuse, and in particular discrimination, more often compared to those with a Dutch background. This confirms earlier research reporting that women of colour in the USA experience more disrespectful care by health care providers during labour and birth compared to white women [11]. In 2020 there was some discussion in Dutch media regarding this subject [39] and the Dutch Federation of Midwives released a statement on eliminating discrimination [40]. However, there are no previous data on the prevalence of discrimination in maternity care in the Netherlands, substantiating the need for further research into this subject.

Higher educated women more often reported not being involved in decision-making compared to less educated women. Leite et al. (2020) found that the higher a women’s educational level, the greater the reported percentage of experienced disrespect and abuse [41]. However, it has also been reported that women with a higher education experience more autonomy in decision making and are more often asked for permission and informed consent [42, 43]. It is possible that women with a higher educational level are more aware of their rights and recognize situations in which they are not fully informed or involved more easily.

For all categories, women who had a subsequent birth experienced upsetting disrespect and abuse less often compared to women who gave birth for the first time. This is in line with previous studies [11, 31, 44–46]. A subsequent birth is generally quicker and more often uncomplicated than a first birth, and therefore there are fewer opportunities for disrespect and abuse to occur. Furthermore, multiparous women know what to expect and have higher confidence levels compared to primiparous women [47]. They are also older on average, and age was also found to be a (mild) protective factor against experiencing upsetting disrespect and abuse in some categories, in line with previous findings [48].

### Strengths and limitations

This study has several strengths. We directly measured experienced disrespect and abuse during labour and birth in the Netherlands from the women’s point of view. It uniquely not only examines the occurrence of situations categorised as disrespect and abuse, but also whether women found these situations to be upsetting. We recruited various organizations and social media influencers to help with data collection, which resulted in a large response; over 13.000 women participated, providing a profound insight in the occurrence of disrespect and abuse among women who gave birth in the Netherlands.

This study also has some limitations. The data of this study are not based on direct observations of disrespect and abuse, but on the perceptions of the women undergoing it. Objective measurement of disrespect and abuse is challenging due to normalization and subjective ways of interpreting situations, which can lead to either under- or overrepresentation of the problem [49, 50]. These complexities should be taken into account while interpreting the findings of the current study. We recommend further research to gain in-depth understanding of women’s experiences and emotions regarding disrespect and abuse during labour and birth, as well as the perspective of the care provider. Nevertheless, this study sheds light on the occurrence of disrespect and abuse as experienced and found upsetting by women and we consider their perspective as legitimate and valuable in its own right.

We focused on the association between respondents’ characteristics and experienced upsetting disrespect and abuse. Although it is relevant to know if disrespect and abuse is associated with birth characteristics such as location or mode of birth, it should be emphasized that, regardless of when, where or how women give birth, we should aim for all births to be free from experiences of disrespect and abuse. Furthermore, previous studies show that it is most often not the intervention itself but rather the interaction around it that influences women’s experience most [15, 51]. Thus, rather than shifting the focus to location or circumstances, we focused solely on the *occurrence* of disrespect and abuse and on the characteristics of the women who suffered from it the most.

The ethnicity of the respondents was based on country of birth of both the respondent and her parents. The classification for this variable was based on CBS Statistics Netherlands, an organization which recently distanced itself from classifying people with a migrant background as Western or non-Western because it is currently seen as debatable and polarising, especially when the respondents’ perspective on the classification of her own and/or her parents country of birth is unknown. Information such as self-identified race or ethnicity would allow a more specific classification, however this information was not available. Therefore, we aimed to present the variable ethnicity as based on country of birth, which is as objective as possible.

Although 101 nationalities were represented among the respondents, women with a migrant background were underrepresented in the study. Recruiting social media influencers and organizations linked to migrant women helped to reach a diverse group of women. However, not all of those who were approached were willing to share the survey with their followers. Also, the questionnaire was only available in English and Dutch, limiting the participation of women who do not read or write these languages or have difficulty to do so. Furthermore, the COVID-19 pandemic made it more difficult to reach women offline. This might have caused women with a migrant background to be less likely to find the questionnaire, which could have influenced the results of this study.

The study population consisted of more primiparous than multiparous women. As the latter experience disrespect and abuse less often, it is possible there is an overrepresentation of experiences of upsetting disrespect and abuse due to parity in this study.

Almost all the characteristics of the women in our sample differed statistically significant from the reference data. However, some differences were quite small, for example ‘singleton or multiple pregnancy’, which only differed 0.5% from the reference data. It is possible some of the characteristics’ statistically significant differences are due to the large sample size of the study [52].

## Conclusions

This study shows women in the Netherlands encounter disrespect and abuse during labour and birth in various forms, with over one third experiencing at least one form of upsetting disrespect and abuse. These upsetting experiences are found to be associated with a more negative labour and birth experience, showing that negative encounters can have major impact on labouring women.

Lack of communication, support and choices were most frequently reported and often perceived as upsetting by women. We argue that these forms of disrespect and abuse should therefore not be seen as light or ‘subtle’. As preferences in communication, support and choices highly depend on women’s personal wishes, these themes require special attention and should preferably already be discussed during antenatal visits.

Although most experienced disrespect and abuse was related to above mentioned categories, physical and verbal abuse were experienced as well. Precisely because these interactions are in all probability not intended as such, care providers need to be aware that women may perceive their actions differently than intended. Teaching programmes should therefore focus more on the emotional aspects of care provision during labour and birth, especially considering the intensity of these events.

Although the occurence of disrespect and abuse is a complex issue and its measurement subjective, this study foregrounds women’s experiences to show that there is still a long way to go before achieving optimal respectful maternity care for all women, even in high income countries.

## Supplementary Information

**Additional file 1: **Additional information on sampling techniques.

**Additional file 2: **Information about the questionnaire development.

**Additional file 3: **Overview of the questions divided in seven categories with answer options yes/no.

**Additional file 4: **Table with an overview of variables.

**Additional file 5: **The respondents’ place of residence at time of birth compared to the national data.

**Additional file 6: **The association between characteristics and upsetting disrespect and abuse during labour and birth: outcomes of univariable and multivariable logistic regression.

## Data Availability

The datasets used and/or analysed during the current study are available from the corresponding author on reasonable request.

## Abbreviations

AOR: Adjusted odds ratio
CBS: Statistics Netherlands
PTSD: Posttraumatic stress disorder
RMC: Respectful maternity care
UMC: University Medical Centre
