# Supplementary material for: Disrespect and abuse during labour and birth amongst 12,239 women in the Netherlands: a national survey
Source: Reprod Health. 2022 Jul 8;19:160. doi: 10.1186/s12978-022-01460-4 (PMC9266084; doi:10.1186/s12978-022-01460-4)
Supplement: Supplementary file 1 — Additional file 1: Additional information on sampling techniques. [file 12978_2022_1460_MOESM1_ESM.docx]

**Additional file 1: Additional information on sampling techniques.**

Social media influencers

Social media influencers are individuals or groups of individuals who shape audiences’ attitudes through online social media channels.[1] Social media influencers often build personal social media profiles in which they express their interests and opinions; can have a significant number of followers, are often commercial actors and sometimes gain (online) fame.[2]

58 Social media influencers who gave birth in the last five years were approached through email or Instagram for their help in disseminating the questionnaire. The approached influencers varied in terms of age, ethnicity, educational level and relational status to reach a diverse population. Variation in pregnancy and birth characteristics was also sought: we approached influencers who (recently) gave birth for the first time, already gave birth multiple times or gave birth to twins. Furthermore, diversity in mode of birth and location of birth was also taken into account, as well as their expressed birth experience (positive and negative).

Sixteen influencers agreed to help with disseminating the questionnaire. Eight influencers had an Instagram account focussed on being a mother; the majority of their posts were related to pregnancy, birth, motherhood and/or family life. The other influencers had a general Instagram account, not directly related to being a mother, who became/had recently become pregnant and shared (some of) their experience online. For example, the influencer was a Dutch celebrity, model or vlogger with an already high number of followers pre-pregnancy.

There were several ways the participating influencers reached their followers, of which two were most common: (1) posting an Instagram story with a ‘swipe-up’ function to the questionnaire. This type of post lasts 24 hours on Instagram and is then automatically removed; (2) publishing an Instagram post with a caption, combined with (referring to) the link of the questionnaire. In the latter, Instagram users can openly respond below the post, allowing ‘user tagging’ to take place, a mechanism in which an Instagram user places a ‘@’ followed by the username of someone they know in order to inform them about the content they just found.[3] This results in a wider dissemination as not only followers of the influencers are reached, but also the individuals that are being tagged.

In the Instagram story or published post, participating influencers asked their followers if they were willing to fill out the questionnaire. Some influencers underlined the importance of the study by sharing (a fragment of) their personal birth experience in their post.


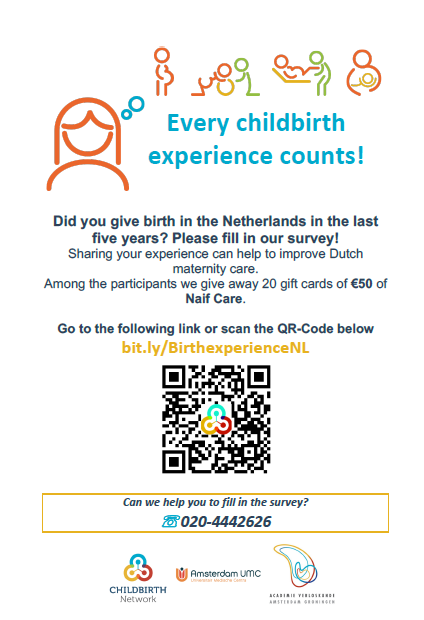
Organizations

17 organizations representing hard to reach groups in society were approached through email or by telephone, of which nine organizations agreed to help disseminate the questionnaire; two midwife practices with a high percentage of women with a non-Dutch background in their practice; one Turkish beauty Saloon, one pregnancy yoga studio, one organization supporting teen moms, one organization supporting single moms, one organization focused on vulnerable women, one organization related to migrant women and one Child and Family Centre in one region in the Netherlands.

The organizations were free to choose how to share the provided content with their members/communities/followers. Options were: (1) sharing the questionnaire in (online) newsletters (2) posting the questionnaire in a closed Facebook group or forum that was associated with the organization (3) sending a separate email to members about the questionnaire (4) Sharing the questionnaire on live occasions. The latter was less often used due to the COVID-19 pandemic.

On the right, an example of a flyer of the study is shown. This material (both in Dutch and English) was used for dissemination purposes by most participating organizations.

*References*

1. Freberg K, Graham K, McGaughey K, Freberg LA. Who are the social media influencers? A study of public perceptions of personality. Public Relations Review. 2011;37(1):90-2. doi: 10.1016/j.pubrev.2010.11.001. PubMed PMID: WOS:000287547100017.

2. Hudders L, De Jans S, De Veirman M. The commercialization of social media stars: a literature review and conceptual framework on the strategic use of social media influencers. Int J Advert. 2020. doi: 10.1080/02650487.2020.1836925. PubMed PMID: WOS:000584836400001.

3. Kang J, Yoon J, Han J, editors. Why do Instagram Users Tag Friends in Comments? Companion Proceedings of the Web Conference 2020; 2020.
