# Supplementary material for: Disrespect and abuse during labour and birth amongst 12,239 women in the Netherlands: a national survey
Source: Reprod Health. 2022 Jul 8;19:160. doi: 10.1186/s12978-022-01460-4 (PMC9266084; doi:10.1186/s12978-022-01460-4)
Supplement: Supplementary file 2 — Additional file 2: Information about the questionnaire development. [file 12978_2022_1460_MOESM2_ESM.docx]

**Additional file 2: Information about the questionnaire development.**

The questionnaire was composed and extended in multiple feedback rounds by the interdisciplinary project team, and then piloted in three rounds among the project team, client representatives with different kinds of birth experiences (to test the content of the questionnaire) and research assistants (to test the routing and flow of the questionnaire). The pilot was used to establish face and content validity. The categories and questions were adapted to the Dutch context and adjusted based on the feedback rounds. Each question covered a certain situation that could have occurred during labour and birth.

The questionnaire was checked by the language monitor unit of the Amsterdam UMC who provided advice on language use, hereby securing comprehension of the questionnaire. The questionnaire was translated by an official agency to secure high quality translation in English. After every test round or check, the questionnaire was adjusted, further composed and extended by the project team and then built in Survalyzer, an online survey software program (Survalyzer Nederland B.V, Utrecht, The Netherlands). Subsequently, the questionnaire was prepared for data collection.
