# Supplementary material for: Disrespect and abuse during labour and birth amongst 12,239 women in the Netherlands: a national survey
Source: Reprod Health. 2022 Jul 8;19:160. doi: 10.1186/s12978-022-01460-4 (PMC9266084; doi:10.1186/s12978-022-01460-4)
Supplement: Supplementary file 3 — Additional file 3: Overview of the questions divided in seven categories with answer options yes/no. [file 12978_2022_1460_MOESM3_ESM.docx]

**Additional file 3: Overview of the questions divided in seven categories with answer options yes/no.**

| **Emotional pressure** |
| --- |
| Were you threatened with bad test results or poor outcomes related to the health of your child? |
| Were threats made that involved withholding care from you or your child? |
| Were you threatened with legal consequences (for example: a child protective services report, criminal charges or other legal proceedings?) |
| **Unkindness/verbal abuse** |
| Did a health care provider say you were overreacting or you were pretending things were worse than they really were? |
| Were you subjected to insulting, harsh, unpleasant and/or derogatory comments? |
| Were you spoken to or shouted at in a harsh/rough or crude/coarse way? |
| Were you verbally abused? |
| **Harsh or rough treatment/physical violence** |
| Were you forced to stay in bed?* |
| Were you forced into a particular position, or were you manually restrained?* |
| Were you subject to rough physical treatment? (for example: pushing, pulling, pinching/gripping, pushing your legs in a particular position) |
| Were you slapped or kicked? |
| Was a (medical) intervention performed that you experienced as physical abuse? |
| Was a (medical) intervention performed that you experienced as sexual abuse? |
| **Lack of communication** |
| Did you feel you were not being involved in the decision-making during labour and birth? |
| Did you feel you were not being listened to? |
| Did you feel you were not being taken seriously? |
| Did you feel insufficiently at ease to ask questions? |
| Did you feel that you weren’t being given information that you should have been given? (for example: not being given full information about what was going on; not being informed about risks and benefits; non-disclosure of test results or diagnoses; not being given information about the progress of labour, not being provided with alternative options). |
| **Lack of support** |
| Did you feel you received too little attention, or were you left alone when you did not want to be left alone? |
| Did a health care provider refuse to assist you? (for example: assistance with going to the bathroom; help with taking a shower; help with managing contractions) |
| Did you ask for pain relief and was your request either ignored or refused by the care provider without there being a clear reason for this? (e.g. pain relief during labour or a local anaesthetic during suturing) |
| Were you or your partner denied (physical) contact with your child, without a clear reason? |
| Did you experience a lack of privacy? (for example during a physical examination) |
| **Lack of choices** |
| Were you not free to decide who would be present at your delivery? (other than health care providers)** |
| Did you feel compelled to accept care that you didn’t really want? |
| Were you not free to decide your position during contractions?* |
| Were you not free to decide the position in which you gave birth?*** |
| Were you told there were certain things you weren’t allowed to do, without there being a clear reason for this decision? (for example: not being allowed to make noise, no eating, drinking, walking around, taking a shower) |
| Was a (medical ) intervention done without your having given clear permission in advance? (for example: a vaginal examination, breaking your waters, performing an episiotomy; administering an injection after the birth) |
| Was a (medical) intervention continued even after you asked for it to be stopped? (for example: being restrained during a vaginal examination) |
| **Discrimination** |
| Did you experience discrimination based on race, ethnicity, cultural background, or language? |
| Did you experience discrimination based on age? |
| Did you experience discrimination based on sexuality and/or gender identity? |
| Did you experience discrimination based on physical or mental disability, illness or complaint? |
| Did you experience discrimination based on religion or belief? |
| Did you experience discrimination based on your appearance (other than racial appearance)? |
| Did you experience discrimination based on education, class, income or other socio-economic factors? |

*Respondents who had a planned caesarean section excluded
**Respondents who gave birth during COVID-19 pandemic excluded
***Respondents who had an epidural, vacuum extraction or a caesarean section excluded
