# Supplementary material for: Disrespect and abuse during labour and birth amongst 12,239 women in the Netherlands: a national survey
Source: Reprod Health. 2022 Jul 8;19:160. doi: 10.1186/s12978-022-01460-4 (PMC9266084; doi:10.1186/s12978-022-01460-4)
Supplement: Supplementary file 4 — Additional file 4: Table with an overview of variables. [file 12978_2022_1460_MOESM4_ESM.docx]

**Additional file 4: Table with an overview of variables.**

| **Variable** | **Categories** | **Additional information** |
| --- | --- | --- |
| **Respondent characteristics** | | |
| Age at time of birth | Mean [SD] |  |
|  | <25 |  |
|  | 25-29 |  |
|  | 30-34 |  |
|  | 35-39 |  |
|  | >40 |  |
| Ethnicity | Both parents are born in the Netherlands | Both parents born in the Netherlands, respondent born in the Netherlands or abroad |
|  | Respondent and (one of) her parents born abroad | (one of ) the parents born in a country other than the Netherlands, the respondent is also born in another country than the Netherlands. |
|  | Respondent born in NL, (one of) her parents born abroad | (one of ) the parents born in a country other than the Netherlands, the respondent is born in the Netherlands |
| Educational level at time of birth | Low | Primary school, first three years of secondary school, or lower level of vocational training |
|  | Middle | Upper secondary school or higher vocational training |
|  | High | Bachelor/master or doctoral degree programs |
| Parity | First birth |  |
|  | Second birth or more |  |
| **Pregnancy and birth characteristics** | | |
| Singleton or multiple pregnancy | Singleton pregnancy |  |
|  | Multiple pregnancy |  |
| Responsible care provider  at onset of pregnancy | Midwife-led care |  |
|  | Obstetrician-led care |  |
|  | General practitioner |  |
|  | Other | Respondents who did not know they were pregnant until (close to) birth, respondents who indicated they did not have a responsible care provider at onset of pregnancy and respondents who indicated they started care abroad (but gave birth in the Netherlands). |
| Planned place of birth one month prior to birth | Midwife-led care at home |  |
|  | Midwife-led care at birth centre |  |
|  | Midwife-led care at the hospital |  |
|  | Obstetrician-led care at the hospital |  |
|  | Hadn’t decided yet |  |
| Birth plan prepared | Yes |  |
|  | No |  |
| Responsible care provider  at onset of labour | Midwife-led care |  |
|  | Obstetrician-led care |  |
|  | General practitioner |  |
|  | No care provider | Respondents who did not know they were pregnant until (close to) birth, respondents who indicated they did not have a responsible care provider at onset of pregnancy |
| Onset of labour | Spontaneous |  |
|  | Spontaneous rupture of membranes, followed by an induction with tablets or oxytocin |  |
|  | Rupture of membranes to induce labour |  |
|  | Induction of labour with tablets/foley catheter/oxytocin |  |
|  | Caesarean section |  |
| Unplanned transfer from midwife-led care to obstetrician-led care | Yes |  |
|  | No |  |
| Mode of birth | Spontaneous vaginal birth |  |
|  | Spontaneous vaginal birth with episiotomy |  |
|  | Vacuum or forceps delivery |  |
|  | Attempted vacuum or forceps, followed by caesarean section |  |
|  | Unplanned caesarean section |  |
|  | Planned caesarean section |  |
| Pharmacological pain relief | No pain relief |  |
|  | Epidural |  |
|  | Remifentanil |  |
|  | Epidural and remifentanil |  |
|  | Other | Gas and Air (Entonox), Pethidine/Morphine |
|  | Epidural and ‘other’ |  |
|  | Remifentanil and ‘other’ |  |
|  | Epidural, remifentanil and ‘other’ |  |
| Anaesthesia during caesarean section | Spinal, epidural or combined spinal/epidural (CSE) |  |
|  | General anaesthesia |  |
| Actual place of birth | Midwife-led care at home | Includes women who gave birth at home before the midwife arrived |
|  | Midwife-led care at birth centre |  |
|  | Midwife-led care at the hospital |  |
|  | Obstetrician-led care at the hospital |  |
|  | Other | On the way to the hospital, home without care provider by choice |
| Gestational age at birth (weeks+days) | <37+0 |  |
|  | 37+0 - 38+6 |  |
|  | 39+0 - 40+6 |  |
|  | 41+0 - 41+6 |  |
|  | >42+0 |  |
| Before or during COVID-19 pandemic | 2015 – February 2020 | Prior to COVID-19 pandemic |
|  | March – December 2020 | During COVID-19 pandemic |
| Care providers present during birth | Community midwife | Includes general practitioners |
|  | Hospital based midwife |  |
|  | Obstetric registrars and/or obstetrician |  |
|  | Maternity care assistant (kraamzorg) |  |
|  | Nurse |  |
|  | Paediatrician |  |
|  | Anaesthesiologist |  |
|  | Care provider in training/student |  |
|  | Other | Doctors from other specialties, paramedics, lactation consultants, ‘assistants’ and ‘surgery team’. |
| Other individuals present during birth | Partner |  |
|  | Other parent(s) of baby (non-partners) |  |
|  | Mother (in law) |  |
|  | Father (in law) |  |
|  | My child(ren) |  |
|  | My sister(s) |  |
|  | My brother(s) |  |
|  | Other family members | Includes niece, aunt, grandma |
|  | Friend(s) | Including neighbors |
|  | Doula | Including labour coach and doula in training |
|  | Birth photographer |  |
|  | Others in care capacity | Sign language interpreter, acupuncturist |
