# Supplementary material for: Disrespect and abuse during labour and birth amongst 12,239 women in the Netherlands: a national survey
Source: Reprod Health. 2022 Jul 8;19:160. doi: 10.1186/s12978-022-01460-4 (PMC9266084; doi:10.1186/s12978-022-01460-4)
Supplement: Supplementary file 5 — Additional file 5: The respondents’ place of residence at time of birth compared to the national data. [file 12978_2022_1460_MOESM5_ESM.docx]

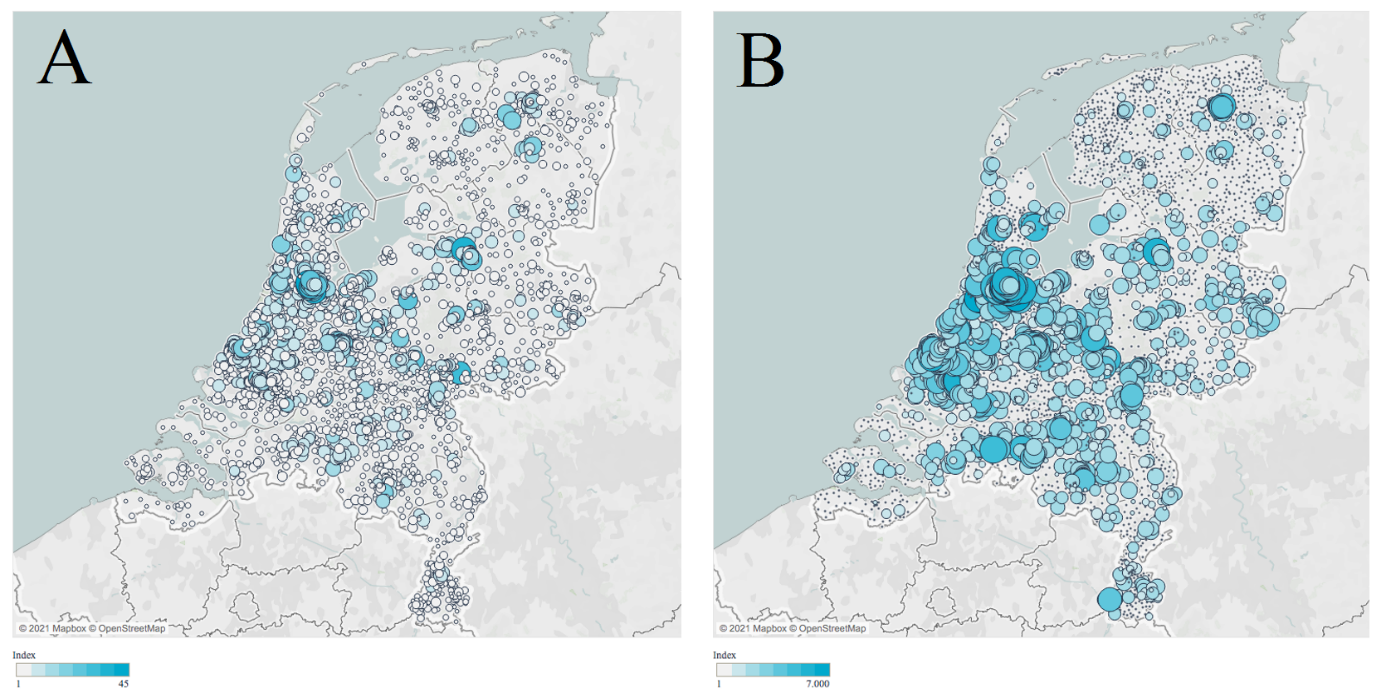
**Additional file 5: The respondents’ place of residence at time of birth compared to the national data.** Distribution of postcodes, with map A representing the respondents’ residence at time of birth (n=12,239), compared with map B of postcodes representing Dutch women aged 15-55 in 2019 (n= 4 317 000).
