# Supplementary material for: Disrespect and abuse during labour and birth amongst 12,239 women in the Netherlands: a national survey
Source: Reprod Health. 2022 Jul 8;19:160. doi: 10.1186/s12978-022-01460-4 (PMC9266084; doi:10.1186/s12978-022-01460-4)
Supplement: Supplementary file 6 — Additional file 6: The association between characteristics and upsetting disrespect and abuse during labour and birth: outcomes of univariable and multivariable logistic regression. [file 12978_2022_1460_MOESM6_ESM.docx]

**Additional file 6: The association between characteristics and upsetting disrespect and abuse during labour and birth: outcomes of univariable and multivariable logistic regression.** Results presented in (1) pooled unadjusted odds ratio [95%CI], (2) pooled adjusted odds ratio [95%CI] and (3) complete case adjusted odds ratio [95%CI].

| **Characteristics** | | | **Pooled* unadjusted odds ratio [95%BI]** | **Pooled* adjusted Odds Ratio [95% CI]** | **Complete case analysis adjusted Odds Ratio [95%CI]** |
| --- | --- | --- | --- | --- | --- |
| **Emotional pressure** | | |  |  |  |
| Higher age | | | 0.99 [0.96-1.02] | 0.990 [0.96-1.02] | 0.99 [0.96-1.02] |
| Ethnicity | Both parents are born in the Netherlands | | 1(ref) | 1(ref) | 1(ref) |
|  | Respondent and (one of) parents born abroad | | **2.10 [1.29-3.41]** | **2.05 [1.26-3.34]** | **2.11 [1.31-3.42]** |
|  | Respondent born in NL, (one of) parents born abroad | | **1.47 [1.02-2.11]** | **1.46 [1.02-2.09]** | 1.44 [0.99-2.09] |
| Educational level | Low | | 1(ref) | 1(ref) | 1(ref) |
|  | Middle | | 0.90 [0.50-1.59] | 0.91 [0.51-1.62] | 0.88 [0.50-1.54] |
|  | High | | 1.19 [0.70-2.01] | 1.23 [0.72-2.10] | 1.16 [0.69-1.96] |
| Parity | First birth | | 1(ref) | 1(ref) | 1(ref) |
|  | Second birth or more | | **0.63 [0.50-0.80]** | **0.65 [0.50-0.83]** | **0.64 [0.49-0.83]** |
| **Unkindness/verbal abuse** | | |  |  |  |
| Higher age | | | **0.97 [0.95-0.98]** | 0.99 [0.97-1.00] | 0.99 [0.97-1.01] |
| Ethnicity | Both parents are born in the Netherlands | | 1(ref) | 1(ref) | 1(ref) |
|  | Respondent and (one of) parents born abroad | | **1.52 [1.09-2.11]** | **1.53 [1.09-2.14]** | **1.60 [1.16-2.21]** |
|  | Respondent born in NL, (one of) parents born abroad | | **1.36 [1.09-1.71]** | **1.35 [1.08-1.70]** | **1.36 [1.09-1.70]** |
| Educational level | Low | | 1(ref) | 1(ref) | 1(ref) |
|  | Middle | | 1.24 [0.90-1.72] | 1.23 [0.90-1.74] | 1.24 [0.89-1.72] |
|  | High | | 1.139 [0.84-1.55] | 1.19 [0.87-1.62] | 1.17 [0.85-1.60] |
| Parity | First birth | | 1(ref) | 1(ref) | 1(ref) |
|  | Second birth or more | | **0.53 [0.47-0.62]** | **0.56 [0.48-0.64]** | **0.53 [0.45-0.62]** |
| **Harsh or rough treatment/physical violence** | | |  |  |  |
| Higher age | | | **0.97 [0.95-0.98]** | **0.98 [0.97-0.99]** | 0.99 [0.97-1.00] |
| Ethnicity | Both parents are born in the Netherlands | | 1(ref) | 1(ref) | 1(ref) |
|  | Respondent and (one of) parents born abroad | | **1.61 [1.24-2.10]** | **1.56 [1.23-2.08]** | **1.55 [1.21-2.07]** |
|  | Respondent born in NL, (one of) parents born abroad | | 1.16 [0.95-1.41] | 1.15 [0.95-1.39] | 1.16 [0.95-1.40] |
| Educational level | Low | | 1(ref) | 1(ref) | 1(ref) |
|  | Middle | | 0.90 [0.70-1.16] | 0.91 [0.71-1.18] | 0.88 [0.68-1.13] |
|  | High | | 0.99 [0.78-1.24] | 1.04 [0.82-1.31] | 0.98 [0.77-1.24] |
| Parity | First birth | | 1(ref) | 1(ref) | 1(ref) |
|  | Second birth or more | | **0.53 [0.48-0.60]** | **0.56 [0.50-0.63]** | **0.54 [0.47-0.61]** |
| **Lack of communication** | | |  |  |  |
| Higher age | | | **0.96 [0.95-0.97]** | **0.98 [0.97-0.99]** | **0.98 [0.97-0.99]** |
| Ethnicity | Both parents are born in the Netherlands | | 1(ref) | 1(ref) | 1(ref) |
|  | Respondent and (one of) parents born abroad | | **1.57 [1.24-1.97]** | **1.57 [1.24-1.99]** | **1.60 [1.27-2.01]** |
|  | Respondent born in NL, (one of) parents born abroad | | 1.17 [1.00-1.37] | 1.16 [0.99-1.37] | 1.17 [0.99-1.37] |
| Educational level | Low | | 1(ref) | 1(ref) | 1(ref) |
|  | Middle | | 1.11 [0.90-1.37] | 1.13 [0.91-1.40] | 1.11 [0.90-1.39] |
|  | High | | 1.11 [0.91-1.35] | 1.18 [0.96-1.44] | 1.16 [0.94-1.42] |
| Parity | First birth | | 1(ref) | 1(ref) | 1(ref) |
|  | Second birth or more | | **0.45 [0.41-0.49]** | **0.47 [0.42-0.52]** | **0.45 [0.41-0.50]** |
| **Lack of support** | | |  |  |  |
| Higher age | | | **0.97 [0.95-0.98]** | 0.99 [0.97-1.00] | 0.99 [0.97-1.00] |
| Ethnicity | Both parents are born in the Netherlands | | 1(ref) | 1(ref) | 1(ref) |
|  | Respondent and (one of) parents born abroad | | **1.34 [1.04-1.73]** | **1.33 [1.03-1.72]** | **1.33 [1.03-1.70]** |
|  | Respondent born in NL, (one of) parents born abroad | | **1.21 [1.03-1.43]** | **1.21 [1.02-1.43]** | **1.22 [1.03-1.45]** |
| Educational level | Low | | 1(ref) | 1(ref) | 1(ref) |
|  | Middle | | 1.00 [0.81-1.25] | 1.01 [0.81-1.26] | 1.02 [0.81-1.28] |
|  | High | | 0.995 [0.81-1.22] | 1.03 [0.83-1.27] | 1.033 [0.83-1.28] |
| Parity | First birth | | 1(ref) | 1(ref) | 1(ref) |
|  | Second birth or more | | **0.47 [0.42-0.52]** | **0.48 [0.43-0.54]** | **0.46 [0.41-0.52]** |
| **Lack of choices** | | |  |  |  |
| Higher age | | | 0.96 [0.95-0.98] | 0.98 [0.97-0.99] | 0.98 [0.97-0.99] |
| Ethnicity | | Both parents are born in the Netherlands | 1(ref) | 1(ref) | 1(ref) |
|  |  | Respondent and (one of) parents born abroad | **1.40 [1.11-1.78]** | **1.40 [1.10-1.77]** | **1.41 [1.11-1.79]** |
|  |  | Respondent born in NL, (one of) parents born abroad | 1.15 [0.97-1.37] | 1.15 [0.97-1.36] | 1.14 [0.96-1.34] |
| Educational level | | Low | 1(ref) | 1(ref) | 1(ref) |
|  |  | Middle | 1.12 [0.89-1.41] | 1.14 [0.90-1.43] | 1.11 [0.89-1.40] |
|  |  | High | **1.25 [1.01-1.55]** | **1.34 [1.07-1.66]** | **1.31 [1.05-1.63]** |
| Parity | | First birth | 1(ref) | 1(ref) | 1(ref) |
|  |  | Second birth or more | **0.50 [0.46-0.56]** | **0.53 [0.48-0.59]** | **0.52 [0.47-0.58]** |
| **Discrimination** | | |  |  |  |
| Higher age | | | **0.93 [0.88-0.99]** | 0.97 [0.93-1.03] | 0.98 [0.92-1.04] |
| Ethnicity | | Both parents are born in the Netherlands | 1(ref) | 1(ref) | 1(ref) |
|  |  | Respondent and (one of) parents born abroad | **5.54 [2.85-10.79]** | **5.93 [3.03-11.60]** | **6.09 [3.12-11.88]** |
|  |  | Respondent born in NL, (one of) parents born abroad | **3.38 [1.95-5.86]** | **3.40 [1.96-5.91]** | **3.33 [1.88-5.88]** |
| Educational level | | Low | 1(ref) | 1(ref) | 1(ref) |
|  |  | Middle | 1.18 [0.52-2.69] | 1.26 [0.55-2.88] | 1.39 [0.57-3.37] |
|  |  | High | 0.51 [0.23-1.15] | 0.55 [0.24-1.26] | 0.61 [0.25-1.47] |
| Parity | | First birth | 1(ref) | 1(ref) | 1(ref) |
|  |  | Second birth or more | **0.52 [0.32-0.83]** | **0.56 [0.34-0.93]** | **0.52 [0.31-0.89]** |

**Pooled data across 20 datasets*
